# Glances at the Hospitals

**Published:** 1901-12-07

**Authors:** 


### GLANCES AT THE HOSPITALS.

### SALISBURY INFIRMARY.

The net income for the year amounted to £4,638, and the total expenditure to £5,509, showing a deficit of £871. Securities to the value of £14,028 have been sold and others bought to the value of £11,257. This left a sum of £2,662, which was applied to the reduction of an adverse balance of £3,618, and the annual income has been increased. It has been decided to enlarge the Herbert Home and to improve the accommodation of the staff at an outlay of £2,500. It is stated that there will be no demand on the funds of the infirmary to meet this expenditure. The Nurses' Home is not yet finished, and although ten years have elapsed since it was decided to build, the funds forthcoming have been totally inadequate. It has therefore been decided to use the Nurses' Superannuation Fund of £1,096 to complete the work, and to replace this when required. The infirmary contained 69 patients at the beginning of the year, and 881 were admitted during it. Of these 446 were discharged cured, 108 relieved, 254 were made out-patients, 29 for other reasons, and 44 died. The daily average number of patients was 83, and of the household 40. The total number of out-patients was 3,007. The diseases are properly classified, and the numbers suffering from the various diseases given, but no details are stated, without which the table is comparatively useless. It is true there is a table showing the causes of death, and from this some information may be fished out. For instance, there were 12 cases of pneumonia, and as no death from this disease occurred, we conclude that all of the 12 cases recovered. There is one case of general paralysis of the insane, but nothing is said about this disease in the death table. What became of the case? On the other hand, there is useful analysis of the surgical operations, and the results carefully tabulated. We notice that 21 amputations were performed without a death. There was one case of trephining, and one death from cerebral compression. The total number of operations was 236, and anæsthetics were used in 222, but, unfortunately, the name of the agent given is not stated.

### SALFORD FEVER HOSPITALS AT LADYWELL AND MODEWHEEL.

During the year 2,144 patients were treated in these hospitals. This number was made up of 181 cases remaining at the end of the previous year, and of 1,963 new cases. Of the total 1,661 were discharged recovered, 255 died, and 228 remained under treatment. The total number treated was far in excess of the average, and Dr. Mullen says that the cubic air space of 2,200 feet originally allowed to each patient had to be encroached on. Two convalescent pavilions are now being erected, and it is hoped that these additions will for the future obviate any necessity for overcrowding. These pavilions will add another line of defence between the public and the recurrence of infectious disease, and they will, as Dr. Mullen points out, play an important part in adding to the material comfort of the patients. The net cost of each patient was £2 18s. 9½d., or 11s. 4½d. per week. The average time spent in the hospitals was 35½ days. We learn that 2,486 lots of bedding and clothing were disinfected at the sanitary station during the year, and it is stated that the horses belonging to the sanatorium travelled a distance of 33,670 miles. It would seem that 36 patients were admitted in a dying state and some of them only survived a few minutes. Deducting these cases the general mortality would fall to 10·4 per cent. of those treated. There were 1,328 cases of scarlet fever. Of these 100 died, showing a mortality of 7·5 per cent. Under one year of age the mortality was 33 3 per cent. among the males and 50 per cent. among the females. Between the ages of 15 and 20 there were only two deaths in 78 cases. Of 216 cases of enteric fever 54 died, being a percentage of 25. The highest number of cases were between the ages of 20 and 25, and here the mortality was 25 per cent. among the males and 17·6 per cent. among the females. The type of disease was a severe one, and it ought to be accentuated that in those received within the first week of the fever the mortality was only 5·8 per cent., while it rose to 60 per cent. in those admitted in the third week, of whom many were suffering from peritonitis, pneumonia, and hæmorrhage. Diphtheria was represented by 294 cases, and of these 71 died, being a mortality of 24·2 per cent. Dr. Mullen says, "The use of anti-diphtheria serum in the treatment of diphtheria has been general and extensive throughout the year, and in the latter part of the year the great advantage to patients of large doses, or of serums of high potentiality in a single dose at the commencement of the treatment, instead of repeated small doses, has been most marked." The report is well arranged and very interesting.

